# Unveiling the Biological Function of *Phyllostachys edulis* FBA6 (*PeFBA6*) through the Identification of the Fructose-1,6-Bisphosphate Aldolase Gene

**DOI:** 10.3390/plants13070968

**Published:** 2024-03-27

**Authors:** Tiankuo Li, Hui Li, Chenglei Zhu, Kebin Yang, Zeming Lin, Jiangfei Wang, Zhimin Gao

**Affiliations:** 1 Key Laboratory of State Forestry and Grassland Administration/Beijing on Bamboo and Rattan Science and Technology, Beijing 100102, China; 2 Institute of Gene Science and Industrialization for Bamboo and Rattan Resources, International Center for Bamboo and Rattan, Beijing 100102, China

**Keywords:** *Phyllostachys edulis*, fructose 1,6-bisphosphate aldolase gene, phylogenetic analysis, protein interaction

## Abstract

Fructose-1,6-bisphosphate aldolase (FBA) is a pivotal enzyme in various metabolic pathways, including glycolysis, gluconeogenesis, and the Calvin cycle. It plays a critical role in CO_2_ fixation. Building on previous studies on the *FBA* gene family in Moso bamboo, our study revealed the biological function of *PeFBA6*. To identify CSN5 candidate genes, this study conducted a yeast two-hybrid library screening experiment. Subsequently, the interaction between CSN5 and PeFBA6 was verified using yeast two-hybrid and LCI experiments. This investigation uncovered evidence that FBA may undergo deubiquitination to maintain glycolytic stability. To further assess the function of *PeFBA6*, it was overexpressed in rice. Various parameters were determined, including the light response curve, CO_2_ response curve, and the levels of glucose, fructose, sucrose, and starch in the leaves of overexpressing rice. The results demonstrated that overexpressed rice exhibited a higher saturation light intensity, net photosynthetic rate, maximum carboxylation rate, respiration rate, and increased levels of glucose, fructose, and starch than wild-type rice. These findings indicated that *PeFBA6* not only enhanced the photoprotection ability of rice but also improved the photosynthetic carbon metabolism. Overall, this study enhanced our understanding of the function of *FBA* and revealed the biological function of *PeFBA6*, thereby providing a foundation for the development of excellent carbon fixation bamboo varieties through breeding.

## 1. Introduction

Glycolysis, also referred to as the Embden–Meyerhof–Parnas (EMP) pathway [1], is an ancient and highly conserved metabolic pathway that predated the existence of oxygen on Earth. This anaerobic catabolic pathway serves two primary functions: oxidizing glucose to generate ATP, reducing agents, and pyruvate, as well as producing components necessary for anabolism [2]. Glycolysis has three distinct features. First, it is the only pathway capable of producing ATP under hypoxic conditions, where pyruvate can be converted into lactic acid. Second, in the presence of sufficient oxygen, pyruvate derived from glycolysis can enter the tricarboxylic acid cycle (TCA). Third, numerous metabolites originating from glycolysis can participate in other metabolic pathways, such as pentose phosphate and glycerol synthesis pathways. These pathways generate essential metabolites such as NADPH and ATP, which are utilized in the synthesis of various substances [1].

The process of gluconeogenesis involves a series of enzymatic reactions that convert non-carbohydrate substrates, such as pyruvate, lactate, glycerol, and specific amino acids, into glucose. The pathway of gluconeogenesis is partially, but not completely, opposite to glycolysis. Since three steps in glycolysis are irreversible, gluconeogenesis requires bypassing these steps and performing the process with a number of characteristic enzymes. Relevant studies have suggested that the EMP pathway exhibits an evolutionary tendency towards glyconeogenesis, as evidenced by phylogenetic evolution and the distribution of enzymes within this pathway [3]. Glycolysis is the breakdown process that converts glucose to pyruvate. In contrast, gluconeogenesis is a synthetic process in which glucose is synthesized from nonsaccharide substances. Certain low-molecular-weight compounds can be converted into hexoses via gluconeogenesis [4].

Notably, the respiratory pathways in plants and animals differ. Animals primarily utilize the fatty acids and pyruvate generated by the glycolytic pathway, whereas plants rarely consume fatty acids for respiration. Therefore, the glycolytic pathway is vital for plants and serves as the principal mechanism driving respiratory metabolism. Moreover, most carbon compounds in the glycolytic pathway and tricarboxylic acid cycle do not produce carbon dioxide but are instead employed in the synthesis of various compounds, such as secondary metabolites, nucleic acids, and amino acids [2].

Glycolysis is a pivotal metabolic process in plants, and its regulation has a significant impact on plant development and stress responses. Recent advances in proteomics and mass spectrometry have revealed extensive dynamic posttranslational modifications (PTMs) in diverse glycolytic enzymes across various plant tissues. These PTMs represent crucial regulatory modifications integrating signal transduction, gene expression, and cellular metabolic networks. Enzymes such as PEP carboxylase (PEPC), cytoplasmic pyruvate kinase (PK), and phosphofructokinases (PFKs) can regulate glycolytic enzyme activity, protein interaction, transient function, and degradation through post-translational modification. Among them, deubiquitination is particularly important [5]. The COP9 signalosome complex (CSN) is a conserved heteromeric protein complex [6], whose primary biochemical function is to debenzylate Cullin-RING E3 ligases (CRLs), thereby regulating these activities [7]. COP9 signalosome complex subunit 5 (CSN5), a part of the CSN, has the function of deubiquitination. FBAs can enhance their stability and promote glycolytic metabolism through deubiquitination [8].

FBA is a vital enzyme in the glycolytic pathway that facilitates the reversible decomposition of fructose 1,6-bisphosphate (FBP) into dihydroxyacetone phosphate (DHAP) and glyceraldehyde-3-phosphate (G3P), which can continue the subsequent glycolytic reactions [9]. Notably, FBA is significant not only in glycolysis and gluconeogenesis but also actively participates in the Calvin cycle [10].

The Calvin cycle, also referred to as the C-3 pathway, operates within the chloroplast matrix of higher plants [11]. This pathway is the primary mechanism for incorporating CO_2_ into carbon compounds during the dark reaction phase of photosynthesis [12]. Comprising 11 enzyme-catalyzed reactions within the chloroplasts, the Calvin cycle utilizes NADP and ATP generated during the photoreaction phase to convert CO_2_ into stable carbohydrates, facilitating the synthesis of starch and sugars [13,14]. It plays a vital role in plant metabolism by supplying intermediates and foundational components for glycolysis, thereby sustaining plant life [15]. The Calvin cycle comprises three main stages: carboxylation, reduction, and regeneration. FBA enzymes contribute to the regeneration phase, where triose phosphate isomerase converts G3P to DHAP, and FBA facilitates the condensation of G3P and DHAP to FBP. Subsequently, ribulose-5-phosphate is generated through a series of catalytic actions involving FBA, sedoheptulose-1,7-bisphosphatase (SBPase), transketolase (TK), and ribose phosphate isomerase, ultimately contributing to the regeneration of ribulose-1,5-bisphosphate (RuBP).

The irreversible reactions of the Calvin cycle are traditionally considered pivotal steps in regulating the flow of carbon metabolism. Previous studies on metabolic regulation have mainly focused on enzymes that catalyze these irreversible reactions [10]. Rubisco, an enzyme that catalyzes the initial irreversible reaction, exhibits notably low catalytic efficiency, thereby historically earning recognition as the principal rate-limiting enzyme in plant photosynthetic carbon assimilation [16]. Hence, enhancing the catalytic efficiency of enzymes through increased activity has become a desirable objective [17,18,19]. However, a metabolic regulation analysis has indicated that a reduction in Rubisco content in transgenic tobacco under normal environmental conditions cannot yield significant changes in the photosynthetic rate [20,21,22]. These findings have suggested that the regulation of photosynthesis by Rubisco can be affected by various factors, such as the plant growth environment and assay conditions. In most environmental conditions, the photosynthetic capacity of plants is not constrained by the level of Rubisco protein [23]. Subsequent investigations have revealed that enzymes responsible for regenerating RuBP can also play a crucial role in governing carbon flux [11,22,24]. Studies on transgenic tobacco, tomato, and rice have suggested that even a slight decrease in SBPase, TK, and FBA catalytic activities can significantly reduce the carbon assimilation rate in antisense transgenic plants and severely hinder plant growth [25,26,27]. Notably, FBA exhibits a carbon flow control coefficient ranging from 0.07 to 0.55 [10,28].

Bamboo is recognized as one of the fastest-proliferating plants globally. A study highlighted significant cell elongation in both the meristematic and elongation zones within bamboo joints compared to other varieties, particularly in the elongation zone [29]. During rapid growth phases, bamboo can simultaneously elongate numerous internodes, sometimes exceeding 40, facilitating efficient DNA replication during cell division [30]. Furthermore, the rapid shoot growth of bamboo involves various physiological processes, including cell division, elongation, and cell wall thickening, all of which require substantial carbohydrate resources. Maternal bamboo plants provide ample non-structural carbohydrates to support the accelerated growth of bamboo shoots through carbon metabolism [31]. Due to the central role of FBA in glycolysis, gluconeogenesis, and the Calvin cycle, exploring the bamboo FBA holds promise for elucidating the mechanisms driving its rapid growth.

In order to identify the biological function of *PeFBA6*, further explore the mechanism of the rapid growth of moso bamboo and lay a scientific foundation for selecting excellent varieties. This study took the following approach to the research: Candidate genes were initially screened from a yeast two-hybrid library and subsequently verified using yeast two-hybrid and LCI experiments. Finally, various physiological parameters were determined, such as the photosynthesis of overexpressed rice.

## 2. Results

### 2.1. Verification of the Interaction between CSN5 and *PeFBA6*

To explore the functional significance of PeFBA6 and uncover its interactions within the glycolysis pathway, we conducted yeast two-hybrid library screening involving PeFBA6. Before the screening, PeFBA6 exhibited little self-activation. Following the screening process on the QDO/X medium, 40 positive clones were isolated. Subsequent bioinformatics analysis contributed to the identification of 25 unique proteins, with CSN5, a participant in deubiquitination modification, being a candidate protein that may interact with PeFBA6 and potentially play a regulatory role in the glycolytic pathway.

**Figure 1:**
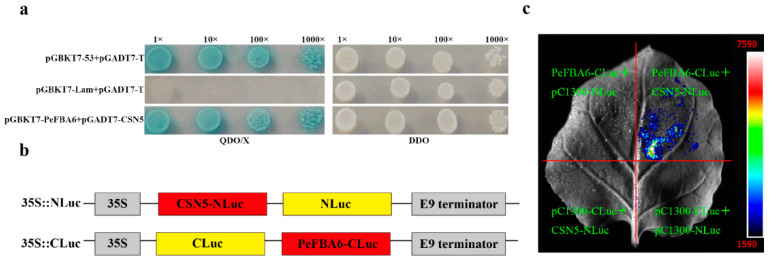
Analysis of the interaction between PeFBA6 and CSN5. (**a**) The interaction between PeFBA6 and CSN5 is validated through a yeast two-hybrid experiment. (**b**) Schematic diagrams of 35S::NLuc and 35S::CLuc constructs (**c**) Additionally, interaction verification between PeFBA6 and CSN5 is conducted using LCI.

The screening results from the yeast two-hybrid library strongly suggested an interaction between CSN5 and PeFBA6. Subsequent validation employed both yeast two-hybrid and firefly luciferase complementation imaging. Co-expressing pGADT7-CSN5 and pGBKT7-PeFBA6 resulted in the robust growth of yeast cells on QDO/X medium, with the yeast turning blue. Nevertheless, no growth was observed in the negative control. Adequate growth was confirmed for pGBKT7-Lam +pGADT7-T on the SD/-Leu/-Trp medium (Figure 1a). To further verify this interaction, 35S::NLuc and 35S::CLuc vectors were constructed (Figure 1b), and the LCI analysis demonstrated a clear interaction between PeFBA6-CLuc and CSN5-NLuc (Figure 1c). These findings provided compelling evidence of the interaction between PeFBA6 and CSN5. In order to clarify the relationship between PeFBA6 and CSN5, an interaction model diagram was made to facilitate understanding (Figure S1).

### 2.2. Determination of Cytoplasmic FBA and Chloroplast FBA Enzyme Activities

**Figure 2:**
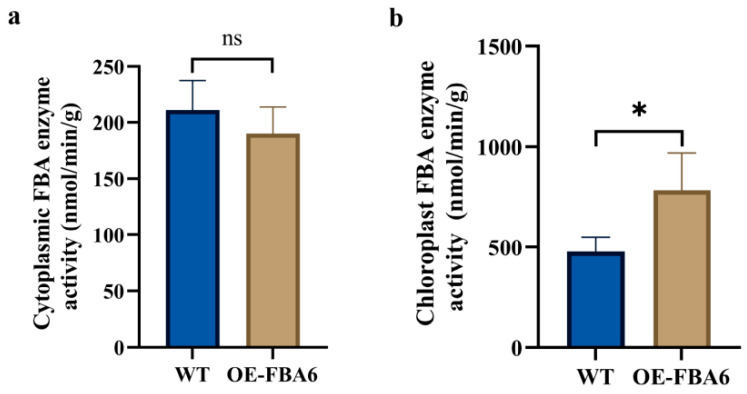
FBA enzyme activity assay. (**a**) Cytoplasmic FBA enzyme activity. (**b**) Chloroplast FBA enzyme activity. * *p* < 0.05, ns: *p* > 0.05. WT: wild-type. OE-FBA6: overexpression of the *PeFBA6*. Each set of averages was derived from three sets of data.

To enhance the reliability of the subsequent verification experiments, we focused on the functional role of *PeFBA6*. Specifically, the FBA enzyme activity was measured in both overexpressed and WT samples. The results revealed no significant difference in the FBA enzyme activity between both groups (Figure 2a). However, chloroplast FBA enzyme activity in overexpressing rice was significantly higher than that in WT rice, with overexpressed rice exhibiting 63.8% higher activity than WT rice (Figure 2b). The data demonstrated the predominant presence of *PeFBA6* in chloroplasts and suggested its potential significance in influencing the photosynthetic carbon metabolism in plants.

### 2.3. Response Curves of WT and Overexpressed Rice

FBA is a pivotal enzyme in both glycolysis and the Calvin cycle, whereas CSN5 is essential for maintaining glycolytic stability, as confirmed by the previously established interaction between PeFBA6 and CSN5. To further elucidate the role of *PeFBA6* in the Calvin cycle and glycolysis, we conducted light and CO_2_ response curve analyses for both the WT and overexpressed sample groups using an Li-6800 instrument.

**Figure 3:**
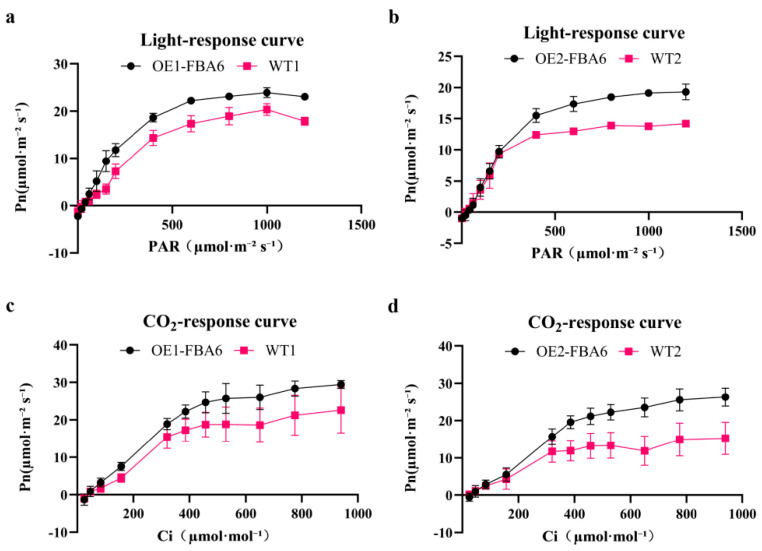
Light response curve and CO_2_ response curve. (**a**) Comparison of light response curves between OE1-FBA6 and WT1 rice. (**b**) Comparison of light response curves between OE2-FBA6 and WT2 rice. (**c**) Comparison of CO_2_ response curves between OE1-FBA6 and WT1 rice. (**d**) Comparison of CO_2_ response curves between OE2-FBA6 and WT2 rice. Pn: net photosynthetic rate. Ci: CO_2_ concentration. PAR: light intensity. WT: wild-type. OE-FBA6: overexpression of the *PeFBA6*. Each parameter was averaged from the six available data sets.

The light response curves reveal different parameters between OE-FBA6 and WT rice. Specifically, OE1-FBA6 and OE2-FBA6 exhibited saturation light intensities of 929.02 μmol·m^−2^·s^−1^ and 982 μmol·m^−2^·s^−1^, respectively. Their maximum net photosynthetic rates were 23.77 μmol·m^−2^·s^−1^ and 19.33 μmol·m^−2^·s^−1^, respectively. The light compensation points for them were 27.92 μmol·m^−2^·s^−1^ and 27.84 μmol·m^−2^·s^−1^, respectively, while their dark respiration rates were 2.66 μmol·m^−2^·s^−1^ and 2 μmol·m^−2^·s^−1^, respectively. In contrast, WT1 and WT2 rice varieties displayed saturation light intensities of 916.09 μmol·m^−2^·s^−1^ and 891.05 μmol·m^−2^·s^−1^, respectively, along with maximum net photosynthetic rates of 20.07 μmol·m^−2^·s^−1^ and 14.19 μmol·m^−2^·s^−1^, respectively. The light compensation points for these two WT varieties were 33.19 μmol·m^−2^·s^−1^ and 23.77 μmol·m^−2^·s^−1^, while their dark respiration rates were 1.52 μmol·m^−2^·s^−1^ and 1.71 μmol·m^−2^·s^−1^, respectively (Figure 3a,b).

The CO_2_ response curve results for OE1-FBA6 and OE2-FBA6 indicate that their respective saturated CO_2_ concentrations were 1309.8·µmol·mol⁻^1^ and 1517.89 µmol·mol⁻^1^. Their maximum carboxylation rates were 53.49 μmol·m^−2^·s^−1^ and 40.23 μmol·m^−2^·s^−1^, while the maximum electron transfer rates were 131.72 μmol·electrons·m^−2^·s^−1^ and 115.88 μmol·electrons·m^−2^·s^−1^. The daily respiration rates were 0.8 μmol·m^−2^·s^−1^ and 0.6 μmol·m^−2^·s^−1^, respectively. In contrast, WT1 and WT2 rice varieties displayed saturated CO_2_ concentrations of 1121.43 µmol·mol⁻^1^ and 1495.73 µmol·mol⁻^1^, along with maximum carboxylation rates of 31.32 μmol·m^−2^·s^−1^ and 30.83 μmol·m^−2^·s^−1^. Their maximum electron transfer rates were 95.77 μmol·electrons·m^−2^·s^−1^ and 67.58 μmol·electrons·m^−2^·s^−1^, while the daily respiration rates were 0.47 μmol·m^−2^·s^−1^ and 0.36 μmol·m^−2^·s^−1^, respectively (Figure 3c,d).

In summary, rice overexpressing *PeFBA6* exhibited significantly elevated respiratory and photosynthetic rates compared with WT rice.

### 2.4. Glucose, Fructose, Sucrose, and Starch Contents

In addition, to investigate the impact of *PeFBA6* on sugar metabolism, leaf samples were collected from both WT and overexpressing rice to assess the levels of glucose, fructose, sucrose, and starch. The sample weight of each group was 0.1 g.

**Figure 4:**
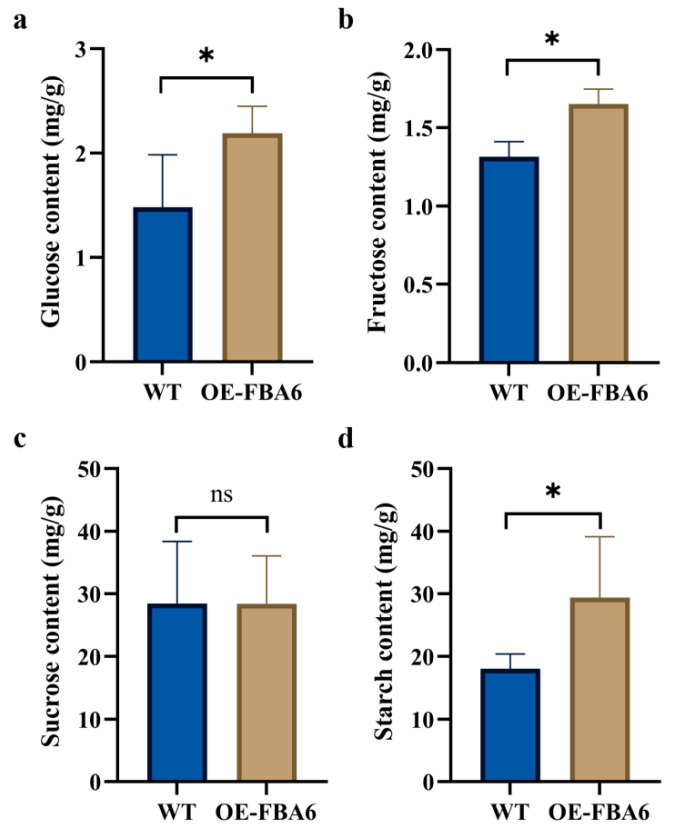
Four kinds of sugar content determination. (**a**) Glucose content. (**b**) Fructose content. (**c**) Sucrose content. (**d**) Starch content. * *p* < 0.05, ns: *p* > 0.05. WT: wild-type. OE-FBA6: overexpression of the *PeFBA6*. Each set of averages was derived from three sets of data.

The results indicated that OE-FBA6 exhibited a 48.11% higher glucose content than the WT (Figure 4a), and a 25.95% higher fructose content than the WT (Figure 4b). However, there was no significant difference in sucrose content between the OE-FBA6 and WT plants (Figure 4c). Notably, the starch content in OE-FBA6 was significantly elevated, surpassing that of WT by 62.8% (Figure 4d). These findings demonstrated that *PeFBA6* enhanced the production of glucose, fructose, and starch, positively influencing sugar metabolism and facilitating rapid growth and carbon fixation in moso bamboo.

## 3. Discussion

Based on prior research on the *FBA* gene family in Moso bamboo, *PeFBA6* was identified as a candidate for the elucidation of biological functions.

Cullin-RING E3 ligases (CRLs) can selectively target specific proteins by incorporating diverse substrate recognition modules (SRMs) for recognizing different substrates [32]. Upon ubiquitin-mediated degradation of the target protein by CRL, the CSN associates with the CRL complex to facilitate its disassembly. This process releases SRM, allowing the CRL complex to be reconstituted with a new SRM [33]. In this study, the interaction between PeFBA6 and CSN5 was confirmed using yeast two-hybrid and LCI experiments. Increased ubiquitin gene expression is correlated with reduced respiratory rates. Enhanced ubiquitinase expression inversely affected FBA expression. In mangoes, elevated ubiquitin expression was observed in affected spongy tissues, accompanied by reduced FBA expression related to glycolysis [34]. In wheat, FBA may interact with F-box, a component of the ubiquitin ligase [35]. According to previous research, aldolase ubiquitination can occur in the cytoplasm [36]. CSN5 possesses a unique catalytic ability to remove the ubiquitin-like Nedd8 modification from CRLs [37]. Relevant studies have indicated that aldolase can enhance its stability through deubiquitination, improving enzymatic activity and the glycolysis rate [8]. Notably, when investigating the genes associated with starch content in tobacco leaves, two potential candidates were identified. One encoded E3 ubiquitin protein ligase, and the other encoded FBA. The results indicated that tobacco plants harboring both genes exhibited higher starch content, implying their potential role in augmenting starch levels in tobacco leaves [38]. This study suggested that FBA in Moso bamboo could undergo cytoplasmic ubiquitination and proposed that CSN5 could enhance FBA protein stability and activity through deubiquitination, ultimately promoting glycolysis rates.

In this study, the *FBA6* gene from Moso bamboo was transferred into the rice genome, resulting in the generation of 30 transgenic rice lines. To ensure the reliability and accuracy of subsequent experiments, the FBA enzyme activities in both WT and overexpressing rice were measured using mixed samples. The findings revealed that chloroplast FBA enzyme activity in OE-FBA6 was 63.8% higher than that in WT. Subsequent experiments were conducted on the basis of the initial experiment. Furthermore, this study verified that *PeFBA6* was potentially a chloroplast gene located in chloroplasts. According to the classification of *FBA* genes, chloroplast FBA is responsible for catalyzing the condensation reaction between FBP and sedoheptulose-1,7-diphosphate within the Calvin cycle [39]. Therefore, the response curve analysis of the *FBA*-overexpressing rice was measured. OE-FBA6 exhibited a higher saturation intensity than the WT, with the WT curve displaying more curvature. This demonstrated that the product of the absorption cross-section of the PS II antenna pigment molecules and the average lifetime of the PS II antenna pigment molecules in the excited state positively corresponded to plant photoinhibition strength [40]. Dark respiration primarily involved glycolysis and tricarboxylic acid cycle pathways. OE-FBA6 displayed a higher dark respiration rate than the WT, indicating increased energy (ATP and NADH) and carbon skeleton production, providing more resources for plant physiological activities. However, dark respiration rates were also significantly decreased during photoinhibition [41]. Typically, the electron transfer rate (ETR) of PS II reflected the photosynthetic electron current size [42], with higher values indicating greater photosynthetic ability. The maximum leaf carboxylation rate, a key parameter affecting plant photosynthetic capacity, played a decisive role in determining photosynthetic rates [43]. In the CO_2_ response curves, OE-FBA6 demonstrated higher maximum carboxylation rates, maximum ETR, and photorespiration rates than WT. These results indicated that *PeFBA6* not only enhanced the photoprotection ability of rice but also elevated the upper limit of the carboxylation reaction in the dark phase, ultimately promoting the photosynthetic carbon metabolism capacity of rice.

The relationship between the glycolytic pathway and the Calvin cycle was integral. The glycolytic pathway decomposes stored carbohydrates during the night while establishing an intermediary shunt during the day to replenish the Calvin cycle [44]. Hence, we assessed the levels of glucose, fructose, sucrose, and starch in the leaves of both overexpressing and WT rice. The results revealed significantly higher glucose, fructose, and starch content in the leaves of overexpressed rice than WT rice, without a notable difference in sucrose content. In plant leaves, the Calvin cycle represented the source, whereas the glycolytic pathway was the energy provider, equating to the sink in the leaves. The Calvin cycle supplied substrate for the glycolytic pathway, and the efficiency of assimilation within the Calvin cycle affected the rate of glycolytic decomposition. Similarly, glycolysis also exerted feedback regulation of the Calvin cycle. The overexpression of the *PeFBA6* gene into rice not only improved the photosynthetic capacity of rice but also elevated its glycolysis rate, thereby providing the material and energy foundation for rice growth and development.

## 4. Materials and Methods

### 4.1. Construction of a Rice Genetic Transformation Vector

Genomic data for Moso bamboo were retrieved from the BambooGDB database (http://bamboo.bamboogdb.org/ accessed on 6 July 2022). The coding sequences (CDS) of *PeFBA6* were cloned and subsequently inserted into the pC1300-UbI-GFP-Flag vector, denoted as pC1300-Flag-PeFBA6, upon successful sequencing. The resulting plasmid was sent to BIORUN BIOSCIENCES, and after a 2-month period, we received both wild-type (WT) rice and *PeFBA6* transgenic rice. A total of 30 transgenic rice seedlings exhibiting positive traits were identified via hygromycin screening. The overexpressed rice was denoted as OE1-FBA6, and OE2-FBA6 and the WT rice was assigned as WT1 and WT2.

### 4.2. Screening of Putative Interaction Proteins of PeFBA6

In this study, the self-activation of PeFBA6 was rigorously examined, revealing no evidence of self-activation. Subsequently, a Y2Hgold monoclone containing the PeFBA6 plasmid was selected from the SD/-Trp (Synthetic Defined Medium without Tryptophan) solid medium and cultured in SD/-Trp liquid medium at 30 °C for 20 h with agitation at 250 rpm until reaching an OD_600_ (Optical Density at 600 nm) of 0.8. The bacterial solution was then centrifuged at 1000× *g* for 5 min. Additionally, 1 mL of AD (pGADT7) bacterial solution and 50 mL of 2× YPDA (Yeast Extract Peptone Dextrose Adenine) liquid medium were added for resuspension. The resuspended solution was transferred to a 2 L conical flask and incubated at 30 °C with constant agitation at 40 rpm for 24 h. Subsequently, the bacterial solution was centrifuged at 1000× *g* for 10 min, resuspended in 50 mL of 0.5× YPDA medium, centrifuged, and resuspended in 10 mL of 0.5× YPDA medium. The bacterial solution was finally spread onto SD/-Trp/-His/-Leu/x-α-gal (Synthetic Defined Medium without Tryptophan, Histidine, and Leucine, supplemented with X-α-Galactosidase, TDO/X) medium and cultured at 30 °C for 5 d. Clones on TDO/X plates were subsequently transferred to SD/-Trp/-His/-Leu/-Ade/x-α-gal (Synthetic Defined Medium without Tryptophan, Histidine, Leucine, and Adenine, supplemented with X-α-Galactosidase, QDO/X) medium for screening. Finally, PCR analysis was performed on the positive clones from the QDO/X plate, and PCR products displaying distinct bands were sequenced.

### 4.3. Yeast Two-Hybrid (Y2H) Assay

The CDS of *PeFBA6* and *CSN5* were cloned and introduced into the pGBKT7 and pGADT7 vectors, respectively. To establish controls, the positive control pGBKT7-53 +pGADT7-T and negative control pGBKT7-Lam +pGADT7-T were prepared according to the Yeast Protocols Handbook (Clontech, Mountain View, CA, USA). The experimental groups containing constructs of pGBKT7-PeFBA6 and pGADT7-CSN5 were co-transformed into the Y2HGold yeast strain (*Saccharomyces cerevisiae*). These co-transformed cells were subsequently cultured on SD solid selection media, specifically SD/-Trp/-Leu (DDO) and QDO/X. The cultures were incubated at 30 °C, and images documenting yeast colony growth were captured.

### 4.4. Imaging of Firefly Luciferase Complementation

The full-length CDS of *PeFBA6* was ligated to the C-terminal, and the full-length CDS of *CSN5* was fused to the N-terminal of the luciferase reporter gene LUC. The resulting constructs were introduced into *Agrobacterium tumefaciens* strain GV3101 (Zoman, Beijing, China). Subsequently, the *Agrobacterium tumefaciens* cultures containing the vectors were co-infiltrated into the tobacco leaves. After 2–3 d, the co-expressed leaves were assessed for LUC activity using a multifunctional imaging system (Tanon, Shanghai, China).

### 4.5. Measurement of the Rice Light Response Curve and the CO_2_ Response Curve

In this study, the Li-6800 (LI-COR, Lincoln, NE, USA) was utilized to measure the light and CO_2_ response curves of both WT and transgenic rice. For each rice genotype, measurements were performed on two leaves per plant. Each leaf underwent multiple measurements until three sets of data with successful fittings were acquired. The final value for each parameter was determined by calculating the average across the six data sets obtained from the two leaves. First, Li-6800 was subjected to self-testing. Within the environmental interface, the flow rate was maintained at 500 μmol/s, with ΔP set to 0.1 KPa. The H_2_O option was configured to maintain a relative humidity (RH) of 50% in the control chamber. For the CO_2_ option, the control CO_2__S was set to 400 ppm. The fan option was adjusted to operate at 10,000 rpm. The temperature settings in the temperature option specified a Tleaf value of 25 °C. Finally, within the light option, the setpoint was set to 600 μmol m^−2^ s^−1^.

In this study, we focused on the central section of the rice leaves for our measurements. Once the parameters in the stability tab reached a stable state, we created an automated program. The intensity gradient was set as follows: 1200 μmol·m^−2^·s^−1^, 1000 μmol·m^−2^·s^−1^, 800 μmol·m^−2^·s^−1^, 600 μmol·m^−2^·s^−1^, 400 μmol·m^−2^·s^−1^, 200 μmol·m^−2^·s^−1^, 150 μmol·m^−2^·s^−1^, 100 μmol·m^−2^·s^−1^, 60 μmol·m^−2^·s^−1^, 40 μmol·m^−2^·s^−1^, 20 μmol·m^−2^·s^−1^, and 0 μmol·m^−2^·s^−1^. The collected data were exported for fitting, and the successfully fitted data were averaged and plotted. The light response curve was fitted using a right-angle hyperbola correction model [45].

CO_2_ response curves were also obtained for the central area of the rice leaves. Once the parameters reached a stable state, an automated program was implemented. The CO_2_ concentration gradient was set as follows: 400 µmol·mol^−1^, 200 µmol·mol^−1^, 100 µmol·mol^−1^, 50 µmol·mol^−1^, 20 µmol·mol^−1^, 400 µmol·mol^−1^, 500 µmol·mol^−1^, 600 µmol·mol^−1^, 700 µmol·mol^−1^, 800 µmol·mol^−1^, 900 µmol·mol^−1^, and 1000 µmol·mol^−1^. After the measurements, the data were processed for fitting, and the successfully fitted data were averaged and presented. The CO_2_ response curve was fitted using the FvCB model [46].

### 4.6. Determination of Glucose, Fructose, and Sucrose Contents

In this study, samples were collected from both WT and overexpressing rice plants in a mixed sampling approach. For each rice variant, four sample groups were designed, with each group weighing 0.1 g. Ultimately, three of these sample groups were selected for inclusion in the calculation of the average values. And the levels of the three sugar types were quantified using a glucose–fructose–sucrose kit (Grace Biotechnology, Suzhou, China). The principle of the kit is as follows: Sucrose and fructose are converted to glucose by the action of specific enzymes, and glucose simultaneously reduces NADP+ to NADPH by the action of enzyme complexes such as hexokinase. The contents of sucrose, glucose, and fructose are calculated by measuring the increase in NADPH at 340 nm.

### 4.7. Determination of Starch Content

The starch content in the leaves of both WT and overexpressing rice was measured using a starch content detection kit (Solarbio, Beijing, China; the sampling method was the same as above). The principle of the kit is as follows: The soluble sugar and starch in the sample can be separated by 80% ethanol, and the starch can be decomposed into glucose by acid hydrolysis. The glucose content can be determined by anthrone colorimetry, and the starch content can be calculated.

### 4.8. FBA Enzyme Activity Assay

The FBA enzyme activity in mixed leaf samples from both WT and overexpressing rice was determined using the plant FBA kit (G0603F, Grace Biotechnology, China; the sampling method was the same as above). The principle of the kit is as follows: FBA can catalyze FBP to G3P and DHAP, and NADH and DHAP to NAD and α-phosphoglycerol under the sequential action of enzymatic complexes. The activity of FBA can be obtained by detecting the decreasing rate of NADH at 340 nm.

### 4.9. Statistical Analysis

Data analysis was conducted using GraphPad Prism 9.5.1 software. To determine significant differences in the data, *t*-tests were employed, with significance denoted as * *p* < 0.05 and nonsignificant results indicated as ns: *p* > 0.05.

## 5. Conclusions

Building upon previous research into the *FBA* gene family in Moso bamboo, this study aimed to elucidate the biological function of the *PeFBA6* gene. Candidate genes were screened through the yeast two-hybrid library and subsequently validated using yeast two-hybrid and LCI experiments. Our results confirmed a protein interaction between PeFBA6 and CSN5, suggesting a potential deubiquitination modification of FBA. Furthermore, *PeFBA6* was overexpressed in rice. The analyses conducted encompassed light response curves, CO_2_ response curves, and glucose, fructose, sucrose, and starch content measurements. Our findings revealed that *PeFBA6* not only enhanced the photoprotection capability of rice but also augmented its photosynthetic carbon metabolism proficiency. These findings can provide a scientific foundation for deeper insights into the *FBA* gene family and the development of superior Moso bamboo varieties through breeding efforts.

## Data Availability

All data generated or analyzed during this study are included in the article and Supplementary Materials.

## Supplementary Materials

The following supporting information can be downloaded at: https://www.mdpi.com/article/10.3390/plants13070968/s1, Figure S1: In order to clarify the relationship between FBA6 and CSN5 more intuitively, a protein interaction model diagram was made in this study.
