# Supplementary material for: Unveiling the Biological Function of Phyllostachys edulis FBA6 (PeFBA6) through the Identification of the Fructose-1,6-Bisphosphate Aldolase Gene
Source: Plants (Basel). 2024 Mar 27;13(7):968. doi: 10.3390/plants13070968 (PMC11013174; doi:10.3390/plants13070968)
Supplement: Supplementary file 1 [file plants-13-00968-s001.zip › plants-2882063-supplementary.pdf]

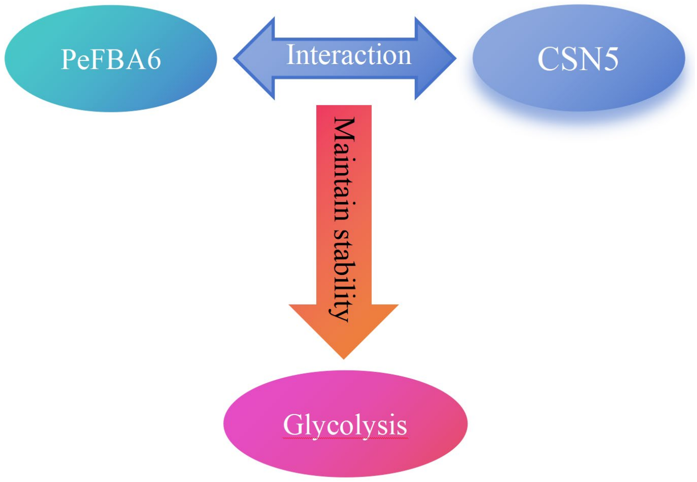

Figure S1. In order to clarify the relationship between FBA6 and CSN5 more intuitively, a protein interaction model diagram was made in this study.
